# Towards a molecular definition of worker sterility: differential gene expression and reproductive plasticity in honey bees

**DOI:** 10.1111/j.1365-2583.2006.00678.x

**Published:** 2006-10-01

**Authors:** G J Thompson, R Kucharski, R Maleszka, B P Oldroyd

**Affiliations:** School of Biological Sciences, University of Sydney Sydney NSW, Australia; * Visual Sciences and ARC Center for the Molecular Genetics of Development, Research School of Biological Sciences, Australian National University Canberra, ACT, Australia

**Keywords:** cDNA microarrays, major royal jelly proteins, Niemann-Pick c proteins, sociogenomics

## Abstract

We show that differences in the reproductive development of honey bee workers are associated with locus-specific changes to abundance of messenger RNA. Using a cross-fostering field experiment to control for differences related to age and environment, we compared the gene expression profiles of functionally sterile workers (wild-type) and those from a mutant strain in which workers are reproductively active (anarchist). Among the set of three genes that are significantly differentially expressed are two major royal jelly proteins that are up-regulated in wild-type heads. This discovery is consistent with sterile workers synthesizing royal jelly as food for developing brood. Likewise, the relative underexpression of these two royal jellies in anarchist workers is consistent with these workers’ characteristic avoidance of alloparental behaviour, in favour of selfish egg-laying. Overall, there is a trend for the most differentially expressed genes to be up-regulated in wild-type workers. This pattern suggests that functional sterility in honey bee workers may generally involve the expression of a suite of genes that effectively ‘switch’ ovaries off, and that selfish reproduction in honey bee workers, though rare, is the default developmental pathway that results when ovary activation is not suppressed.

## Introduction

The expression of altruistic helper traits such as sterility and alloparental care (i.e. provision of care to an individual that is not an offspring) usually comes at a cost to the altruist's direct reproductive success. Despite this direct cost, alleles that increase altruistic behaviour can persist in populations and increase in frequency if their expression in some individuals has the effect of increasing the fitness of others that carry non-expressed replicas (Hamilton, 1964). Thus, one opportunity for identifying ‘genes for altruism’ is via expression-based genomic screens.

In honey bee societies, queens constitute the reproductive female caste and activate their ovaries within a few days of mating. Workers, by contrast, cannot mate and almost never activate their ovaries in the presence of their queen, rendering themselves functionally sterile (Butler, 1957). Thus in honey bees, worker sterility is a function of ovary activation, and genes for sterility might simply encode proteins that suppress ovary activation under certain conditions – as in queenright honey bee workers. If so, these genes could potentially be identified by comparing the gene-expression profiles of queens against workers: genes differentially expressed between queens with active ovaries and workers without them should include those that generate this reproductive difference. This approach is, however, considerably complicated by the fact that queens and workers are strongly differentiated across a vast array of characteristics, including many that are not directly related to reproduction (Michener, 1974; Winston, 1987). As a consequence, differences in gene expression between the two castes are considerable (Evans & Wheeler, 2000). This makes the detection of genes specifically associated with ovary activation, and thus sterility, difficult.

An alternative approach toward the isolation of genes specifically associated with suppression of ovary activation in workers is to compare the expression profiles of ovary-activated and ovary non-activated individuals within this caste. Rare though ovary-activated queenright workers are (Visscher, 1989), genes differentially expressed between them and their sterile sisters are more likely to include genes that directly ‘switch’ ovaries on or off within individuals, as opposed to directing other aspects of caste differentiation.

In this study, we use a series of two-colour cDNA microarrays generated from the bee brain expressed sequence tag (beeEST) project (Whitfield *et al*., 2002) to screen ≈ 5500 genes for expression differences related to ovary activation in developing workers. Our approach was to compare the gene expression profile of young wild-type workers against that of ‘anarchists’, a mutant strain of honey bee in which queenright workers regularly develop into selfish egg layers at high frequency (Oldroyd *et al*., 1994; Oldroyd & Osborne, 1999). Patterns of inheritance of the anarchic and wild-type phenotypes strongly suggest that the anarchic phenotype is ultimately controlled by a small number of genes, possibly as few as two (Montague & Oldroyd, 1998; Barron *et al*., 2001). The availability of the mutant strain makes it possible to screen for genes whose expression is directly associated with the onset of ovary activation, while avoiding differences related to caste, age or social environment. These could include the immediate early gene(s) that regulate ovary activation, and downstream genes that are controlled by these genes. Thus genes differentially expressed between young anarchist (AN) and wild-type (WT) workers are likely to be those that are directly involved in the regulation of worker sterility, or are differentially expressed as a consequence of genes that regulate worker sterility. This screen is an important step toward deconstructing the molecular pathway that regulates functional sterility in honey bee workers.

## Results

**Figure 1:**
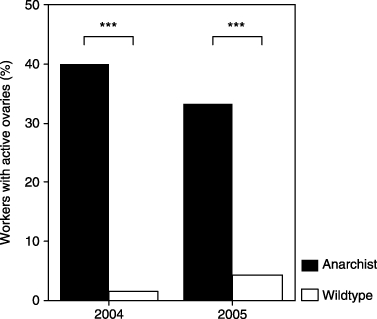
Summary of ovary assay for the two focal colonies; one chosen per year. Anarchist workers have higher levels of ovary activation compared with wild-types. Contingency table analyses indicate that all comparisons are significant (both *P* < 0.001).

Our cross-fostering field experiments yielded co-reared WT and AN workers that showed substantial differences in degree of ovary activation, indicating a strong genetic effect on variation in ovary activation (Fig. 1). In 2004, the greatest difference between WT and AN workers was found in host colony ‘WT5’ (2% vs. 40%; *n* = 129, 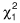 = 24.8; *P* < 0.001). In 2005, the greatest difference between WT and AN was found in host colony ‘WT1’ (4% vs. 33%; *n* = 143, 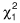 = 17.7; *P* < 0.001). We used these two biological contrasts in our genomic screen.

Hybridization quality plots did not reveal any technical aberrations or systematic biases in the normalized gene expression data (see Supplementary material). Statistical differences inferred from the normalized data should therefore represent differential gene expression and not technical bias.

**Figure 2:**
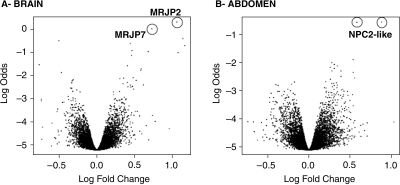
Volcano-plots showing the log-fold change (i.e. *M*) of each gene against the log-odds for differential expression. Positive values on *x*-axis indicate up-regulation in WT; negative values indicate up-regulation in AN. Labelled genes are significantly differentially expressed (*q* < 0.05, see text). MRJP, major royal jelly protein; NPC2-like, Niemann–Pick type C2-like protein.

A volcanoplot showing the statistical distribution of positive vs. negative expression-fold changes reveals a general symmetry in terms of up- vs. down-regulation (Fig. 2), but a tendency for up-regulation in WT is present among the most informative genes of both experiments (brain, abdomen). Among these are four beeESTs that show strong evidence for differential expression. They are BB160004B10E06 and BB170007B20D05 (circled on Fig. 2A) and BB170032A10H06, beeEST BB170008A10E05 (circled on Fig. 2B). The remainder of beeESTs, representing the vast majority of genes screened, fall below the adjusted threshold for significance, and thus show no strict evidence of differential gene expression.

Table 1 shows the top 20 most informative genes, as ranked by the absolute value of *t*-statistics in each experiment. The four beeESTs with significant (*q* < 0.05) differences in their expression between AN and WT workers (Fig. 2) represent two major royal jelly proteins (MRJP) up-regulated in WT worker heads, MRJP2 and MRJP7, and one Niemann–Pick type C2 protein (NPC2-like) up-regulated in WT worker abdomens. Thus, the two beeESTs significantly up-regulated in abdomens correspond to one predicted gene, NPC2-like, yielding a total of three genes differentially expressed. We regard the three differentially expressed genes identified from this analysis as important predictors of ovary activation in honey bee workers. These candidates are strongly associated with reproductive class in developing workers, and show expression fold differences of 2.08-fold (MRJP2), 1.65-fold (MRJP7) and ≈ 1.67-fold (NPC2-like).

**Table 1:** Top-20 most informative genes implicated in the regulation of worker sterility. We present separate lists for brain and abdominal tissue experiments. For each gene we show the log ratio of expression (*M*, see text), where positive values indicate higher expression in the WT, as well as other summary information

Rank	beeEST	Accession	Sign	Log-ratio, *M*	*t*-statistic	*Apis mellifera* gene†	Linkage group‡	Homology
Brain tissue								
1	BB160004B10E06	BI511095	+	1.06266	10.54052*	MRJP2 (AF000632)	LG11	−
2	BB170007B20D05	BI508208	+	0.73022	8.90504*	MRJP7 (NM_001014429)	LG11	−
3	BB160007A10D09	BI511842	+	1.14376	7.31903	MRJP3 (GB15390)	LG11	−
4	BB160021B10H08	BI516199	−	0.50340	−7.03322	Predicted (XM_396605)	−	−
5	BB160019B10C02	BI515491	+	0.44407	6.74209	Predicted (GB11494)	LGUn	CBG03739
6	BB160003B10B11	BI510780	+	1.16874	6.47057	Predicted (GB12564)	LG12	synapsin
7	BB170018B10A09	BI509691	+	0.46876	5.97984	MRJP5 (GB10622)	LG11	−
8	BB170018B20G10	BI506143	+	0.68512	5.89985	MRJP5 (GB10622)	LG11	−
9	BB160022A10G11	BI516332	+	1.07841	5.80371	MRJP3 (XM_391893)	LG11	−
10	BB160012B20G11	BI513504	+	0.46171	5.54909	Unknown	LGUn	−
11	BB170014B20E03	BI505979	−	0.76631	−4.86389	Unknown	LG2	−
12	BB160010A10B11	BI512703	+	0.40742	4.79587	Predicted (GB14785)	LG7	mapmodulin
13	BB160023B20H03	BI516947	+	0.58835	4.58093	MRJP4 (NM_001011610)	LG11	−
14	BB170004A20C08	BI503042	+	0.58392	4.50234	Unknown	LG14	−
15	BB170012B20B02	BI509225	−	0.47082	−4.35128	Predicted (GB11410)	LG4	CG31803
16	BB160006A20B02	BI511564	+	0.48269	4.05937	Unknown	LG1	−
17	BB160016B10G12	BI514886	+	0.35545	4.02584	Predicted (GB16628)	LGUn	RpL6
18	BB170019B20B11	BI506253	+	0.30753	3.99177	Predicted (GB11074)	LG2	CG7886
19	BB160005B10G11	BI511393	−	0.31552	−3.94055	Predicted (GB17176)	LG4	CG7231
20	BB170011B20D04	BI503319	+	0.52384	3.90507	Predicted (GB13722)	LG6	glucocerebrosidase
Abdominal tissue								
1	BB170032A10H06	BI505487	+	0.8815213	11.776712*	Predicted (GB14261)	LG5	NPC2-like
2	BB170008A10E05	BI507942	+	0.5827994	11.635177*	Predicted (GB14261)	LG5	NPC2-like
3	BB160011A10H06	BI513067	+	0.5359440	5.144227	Unknown	−	−
4	BB160014B20G06	BI514297	+	0.2770965	4.905664	Unknown	LGUn	−
5	BB160020A20G08	BI515752	+	0.3076986	4.775538	Predicted (GB17081)	LG11	ubiquitin
6	BB160016B20E02	BI514937	+	0.3445220	4.676179	Predicted (GB14785)	LG7	mapmodulin
7	BB170003A20H02	BI504934	+	0.2577799	4.575170	Unknown	LG5	−
8	BB160017A20E09	BI515062	+	0.3084728	4.496380	Unknown	LG2	−
9	BB160022A20E11	BI516405	+	0.2790998	4.347537	Unknown	LGUn	−
10	BB170011A10B12	BI508145	+	0.3882265	4.346908	Predicted (GB13731)	LG15	RpL26
11	BB170026B10D08	BI509796	+	0.5366856	4.242169	Unknown	LG13	−
12	BB170014B10G12	BI509138	+	0.3886676	4.011500	Predicted (GB17541)	LG15	CG5059
13	BB160022A10F07	BI516320	+	0.5546391	3.919889	Predicted (GB13399)	LGUn	myosin
14	BB160021B20G08	BI516272	+	0.2588436	3.844824	Predicted (GB13198)	LG9	CG14232
15	BB160019A10F04	BI515339	+	0.2025632	3.719943	Predicted (GB19244)	LGUn	MGC89629
16	BB170020B10D08	BI505261	−	0.1759085	−3.701303	Unknown	LG14	−
17	BB160022A10E02	BI516305	+	0.3322998	3.664078	Predicted (GB13621)	LG1	Solute carrier
18	BB160019A20D08	BI515373	+	0.2759870	3.628079	Predicted (GB13198)	LG9	(= to 14)
19	BB170023A10E04	BI509838	+	0.3020104	3.624914	Predicted (GB15437)	LG2	CG17034
20	BB170029B10F04	BI505465	+	0.2807702	3.624723	Predicted (GB15483)	LGUn	RpS19e

* Indicates significance, *q* < 0.05.

† The accession number (GBxxxxx) from the Official Predicted Gene Set (GLEAN3) is provided (BeeBase, http://racerx00.tamu.edu/bee_resources.html), otherwise the GenBank accession number is provided.

‡ By default, in relation to Build 2.1 statistics (http://www.ncbi.nlm.nih.gov/).

Many beeESTs had small (< 0.05) unadjusted *P*-values (*n* = 182 brains; *n* = 262 abdomens), but only four had significant *q*-values. The distribution of *q*-values showed a large gap between the few that were significant (<< 0.03) and the vast majority that were non-significant (> 0.10) values. This discontinuity in *q* distribution indicates that the differentially expressed gene set is clearly distinguished from the constantly expressed (or non-expressed) gene set.

As was apparent from visualizing the probe-level data (Fig. 2), the top-20 most informative genes show a trend towards up-regulation in WTs (Table 1). This pattern is present in brain (16 of 20; 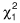 = 2.74, *P* = 0.097) and abdominal (19 of 20; 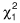 = 8.03; *P* = 0.005) experiments. As expected at only 4 days old, there were no massive differences in gene expression between AN and WT strains. No top-20 genes showed expression differences vastly greater than twofold (i.e. where M >> 1). This tight variance in expression ratio is in contrast to variation in average intensity, which did range more than 10-fold among the top-20 genes: *A*-values ranged from 7.6 to 12.5 for brain, and 8.2 to 11.9 for abdomens (in log_2_ scale).

## Discussion

We have identified a set of three genes that are differentially expressed between WT and AN workers: a Niemann–Pick type C2 homologue and two major royal jelly proteins. NPC2-like is the only gene that is significantly differentially expressed in abdominal tissue, and is up-regulated in WT relative to AN. MRJP2 and MRJP7 are the only genes significantly differentially expressed in head tissue, and are also up-regulated in WT, relative to AN (Table 1).

Our interest is to find genes that are differentially expressed between young WT and AN workers before ovary activation *per se* is apparent. The two strains show strong genetically determined differences in their proclivity to activate ovaries and lay eggs: WTs are functionally sterile, while ANs typically activate their ovaries and lay large numbers of viable eggs (Oldroyd *et al*., 1994; Oldroyd & Osborne, 1999). Genes differentially expressed between young WT and AN workers should therefore include those that regulate the conditional expression of worker sterility. We screened roughly 40% of genes in the honey bee genome (Whitfield *et al*., 2002; Honey Bee Genome Sequencing Consortium, 2006) and found, after applying a strong correction for false positives, a very small proportion of them (< 0.1%) were statistically differentially expressed. Though our screen was not comprehensive, by design it targeted genes specifically associated with onset of conditional expression of worker sterility.

As a general trend, the most informative genes (Table 1; Fig. 2) tended to be up-regulated in WTs. This observation falsifies a null expectation for symmetry of expression data, and suggests that functional sterility in honey bee workers may generally result from the expression of a suite of genes that effectively ‘switch’ ovaries off early in adult development. In normal WT colonies, the environmental cue mediating this switch is the presence of a functional queen and her brood, as signalled (Keller & Nonacs, 1993) by queen and brood pheromone (Hoover *et al*., 2003). Exposure to queen pheromone does strongly affect gene expression in worker brains (Grozinger *et al*., 2003). This ‘genes-on-to-switch-off’ reproduction hypothesis has some additional support, as Evans & Wheeler (2000) noted that as larvae develop into sterile workers they up-regulate many more genes than do larvae developing into reproductive queens. The reproductive state (with ovaries activated) may therefore be the developmental default for both workers and queens. If so, then departures from default would normally be effected by pheromonal cues, or in the case of AN workers, by a mutation that affects the threshold response to such cues (Barron *et al*., 2001; Oldroyd *et al*., 2001).

The differential expression of NPC2-like, MRJP2 and MRJP7 between WT and AN workers is probably symptomatic of a fundamental difference in the reproductive development of these two strains. Though the precise reason for their differential expression in the current study is not yet known, MRJPs are known to mediate reproductive maturation and the expression of honey bee social behaviour at several levels. First, at an ultimate level MRJPs are coevolved with *Apis* eusociality (Drapeau *et al*., 2006a), implying an intimate association between the biological function of MRJP genes, and the expression of social traits, especially alloparental care and sterility. The Yellow gene family from which MRJPs are derived (pfam03022) is widespread within the Arthropoda, but MRJPs are currently unknown beyond the genus *Apis*. It is noteworthy that *A. mellifera*, *A. cerana*, *A. dorsata* and *A. florea* are all highly eusocial (Michener, 1974), and all have genes encoding MRJPs (Albertova *et al*., 2005; Imjongjirak *et al*., 2005; Su *et al*., 2005). Whether the phylogenetic association between sociality and MRJPs is significant has not yet been tested, but the apparent coevolution between the two characters suggests that certain MRJPs evolved to help signal or regulate the expression of social traits, possibly including worker sterility. Some Yellow/MRJP genes do have regulatory roles (Maleszka & Kucharski, 2000) and their expression in the head (or brain) here does suggest a role in behaviour.

Second, and at a proximate level, MRJPs are synthesized by young nonreproductive workers and incorporated into royal jelly (RJ), or ‘brood food’– a major determinant of caste differentiation, and thus a major determinant of an individual's direct reproductive potential. Larvae fed large amounts of RJ beyond a critical period (about 3 days) develop into highly fecund queens, the rest develop into barren workers (Seeley, 1985). It is possible that ANs and WTs differ in their response to this nutritional cue during larval development, with ANs developing queen-like reproductive traits (Beekman & Oldroyd, 2003). This would not, however, explain why 4 day olds reared in a common WT host colony would show expression differences at MRJP2 and MRJP7 loci. A more likely explanation emerges when we consider that 4 day olds are producers of RJ, rather than recipients. The synthesis of RJ by nurse-age workers, and its subsequent provision to the queen and her brood is a form of kin-selected alloparental care. The expression of MRJP2 and MRJP7 in WT workers presumably reflects the typical production of these two MRJPs. They would likely use the synthesized protein to provision the larvae. The relative down-regulation of these two RJ proteins in AN workers, by contrast, reflects the tendency for this strain to generally abandon altruistic helping, including alloparental care (Dampney *et al*., 2004), in favour of selfish egg laying (Barron *et al*., 2001). ANs differ from WTs through a ‘syndrome’ of traits related to ovary activation and worker sterility, including reduced sensitivity to pheromones produced by queens (Oldroyd *et al*., 2001; Hoover *et al*., 2005) and by brood (Oldroyd *et al*., 2001), which generally result in dysfunctional colonies that cannot sustain themselves (Barron *et al*., 2001).

Though probably not a direct primer of ovary deactivation or sterility *per se*, our observation that MRJP2 and MRJP7 are expressed in WTs suggests that the upward expression of these two proteins is at least temporally associated with the conditional expression of sterility. These two genes occur side-by-side in the honey bee genome as part of a tandem series of 10 MRJPs on linkage group 11. MRJPs are likely derived via gene duplication of an ancestral yellow-e3 gene (Drapeau *et al*., 2006a). Their physical linkage and coregulation in the present study suggests that MRJP2 and MRJP7 could be coregulated components in a pathway related to the expression of worker sterility. If MRJPs retain ancestral functions inherited from their Yellow progenitors, then they may well be key players in reproductive development, as they are in flies and ants (Drapeau *et al*., 2006b). In addition, we note the presence of additional MRJP genes among the top-20 set for brains (Table 1), including MRJP3, MRJP4 and MRJP5. In total, this set represents five of the nine protein-encoding genes that make up the MRJP gene family.

The involvement of an NPC2-like gene in a pathway associated with the regulation of worker sterility is harder to assess, because its function in honey bees is not known. None the less, it too seems to play a part in nutrition, and thus could likewise play a part in the mechanics of honey bee sociality (Seeley, 1985). As the only gene significantly up-regulated in abdomens, this protein's NPC2 domain presumably binds lipids and cholesterols in honey bees as it does in flies, mosquitoes and mammals. In humans, mutations at this eponymous locus cause a type of lipid-storage disorder called Niemann–Pick disease (Garver & Heidenreich, 2002). Not previously implicated as important to honey bee reproduction or sociality, the NPC2-like gene identified here, via two experimentally independent differentially expressed beeESTs (Table 1), is a new candidate component for the deactivation of worker ovaries, and thus a new candidate for the regulation of worker sterility in honey bees.

### Towards a molecular definition of worker sterility

The co-occurrence of castes with alloparental care is the essential criterion that defines honey bee eusociality (Michener, 1974). MRJPs are involved in the evolution and expression of both of these traits, and are thus intimately linked to reproductive altruism and indirect reproduction by honey bees. The down-regulation of NPC2-like, MRJP2 and MRJP7 in 4-day-old AN workers suggests that these genes are actively linked to the reproductive status of individuals. Moreover, they appear to be regulated directly or indirectly by the underlying mutation present in the AN strain that causes workers to effectively ignore the normal semiochemical cues for ovary deactivation.

MRJPs are similar to vitellogenin in being proteins important to the evolution of eusociality (Amdam *et al*., 2003, 2006). Like MRJPs, vitellogenin is synthesized by nonreproductive workers and incorporated into RJ (Engels, 1974). Whether ANs overexpress vitellogenin could not be tested in the present study because unfortunately this gene is not represented on the array (Whitfield *et al*., 2002). However, an independent study that used a quantitative polymerase chain reaction (PCR) assay to test the expression of vitellogenin as a function of ovary activation in honey bee workers (Koywiwattrakul *et al*., 2005) found that vitellogenin is overexpressed in ovary activated workers, albeit in abdomens not brains, relative to workers whose ovaries had been experimentally inhibited. It is conceivable therefore that both MRJPs and vitellogenin are part of a single pathway regulating the honey bee reproductive division of labour.

The next step in this line of research is to study these genes in isolation and deduce their function and interdependence. Moreover, though the underlying mutation that caused our candidates to be differentially expressed has not yet been mapped, the candidates do represent new positional targets for future mapping studies. We currently have such studies in progress and, together with functional tests of individual genes, and the derivation of new hypothetical models of gene action, we hope to help deduce the yet-to-be-described molecular pathway that regulates functional sterility in honey bee workers. The future description of this pathway will be of profound theoretical significance.

## Experimental procedures

### Biological material and ovary assay

To obtain biological material for the microarray we incubated sealed brood combs containing emerging adult workers removed from WT (*n* = 2) and AN (*n* = 2) colonies, at 35 °C overnight. The following morning, we paint-marked ≈ 800 adult workers of each genotype of each colony and fostered them into unrelated queenright WT (*n* = 2) and AN (*n* = 2) host colonies, creating a total of 16 within-colony WT vs. AN contrasts. After 4 days we collected a subsample (*n* = 10) of same-colour workers from each host colony and snap-froze them in liquid N_2_ to stabilize their RNA in tissue. Importantly, 4-day-old workers show no sign of ovary activation, and so genes that are differentially expressed between WT and AN workers at this age are not due to the presence of oocytes. Rather we sought genes that are differentially expressed prior to the appearance of eggs as these are likely to be the genes that prevent ovary activation in WT workers. After 16 days, when the proportion of ovaries activated is highest (personal observations), we collected all remaining paint-marked bees and scored their ovaries as activated (visible ova), or not (after Dade, 1977). We assessed differences in ovary activation between WT and AN strains reared within single colonies using contingency table analyses. A second cross-fostering experiment was performed with a different set of WT and AN colonies in the following year.

### RNA extraction

We extracted RNA from each year's (2004, 2005) 4-day-old sample group whose older siblings (same colour and genotype, but allowed to mature to 16 days) showed the greatest difference in ovary activation. We extracted RNA from these individuals from both abdominal tissue and brain tissue separately, using a modified Trizol/Qiagen protocol as described in Koywiwattrakul *et al*. (2005). For abdomens, we extracted RNA from whole abdomens (minus appendages) then pooled standardized aliquots of RNA to yield two composite RNA samples: one representing WT, the other AN. For brain tissue, we pooled dissected (cf. Kucharski & Maleszka, 2003), individual brains prior to the RNA extraction. In each case, we used 500 ng of RNA as input for fluor-labelled cDNA synthesis.

### Fluorescent cDNA synthesis

We synthesized, amplified and fluorescently labelled cDNA using the Low RNA Input Fluorescent Linear Amplification Kit (Agilent Technologies, Palo Alto, CA, USA). We poly(A)+ selected mRNA transcripts and reverse transcribed them into double-stranded cDNAs using a T7 promotor primer and MMLV reverse transcriptase. We then generated unlabelled cRNA from cDNA using T7 RNA polymerase, then purified this amplified product using spin filtration (QIAGEN's RNeasy Mini Kit). We converted 500 ng of cRNA into fluorescently labelled cDNA using MMLV reverse transcriptase, random hexamer primers, and cyanine 3-dCTP (Cy3; 532 nm) or cyanine 5-dCTP (Cy5; 635 nm) fluorescent labelling. We combined alternately labelled cDNA samples, purified them by spin filtration (QIAGEN's QIAquick PCR Purification Kit), and eluted the labelled cDNA mixture in a single 45-litre volume of Qiagen Buffer EB prior to hybridization on to the arrays.

### Comparative genomic hybridizations

To hybridize labelled cDNA on individual arrays we combined 42.5 L eluate (containing Cy3 and Cy5 labelled probes), 7.5 µl 20 × SSC, and 50.0 µl ExpressHyb hybridization buffer (BD Biosciences Clontech, Palo Alto, CA, USA). This 100-litre hybridization mixture was heat-denatured (90 °C, 2 min), centrifuged (13 000 ***g***, 2 min), and immediately dispensed on to the array's glass substrate and covered with a glass slip. We encased each substrate-based reaction within a Corning CMT hybridization chamber, which was then incubated (62 °C, 4–6 h). Following incubation, we performed a series of stringency washes on the arrays (2 × SSC 0.1% SDS; 2 × SSC; 0.1 × SSC) and spun-dried (500 r.p.m., 2 min) each array prior to acquiring fluorescent images of hybridization signal. We used a direct design (cf. Yang & Speed, 2002) for comparative cDNA hybridizations. Specifically, we compared WT and AN mRNAs using two pairs of dye-swap hybridizations, where each dye-swap pair represents a biological replicate obtained in a different pair of colonies in a separate year. This design was applied to brain and abdominal tissue separately. Following background correction and data normalization (described below) we calculated differential expression for each gene as a fold-change, namely, the log-ratio of hybridization signal intensities: *M* = log_2_ (WT/AN).

### Microarray analysis

The arrays used in this experiment were printed from a normalized and subtracted beeEST library. Details of the source library and array manufacture are described in Whitfield *et al*. (2002, 2003). We scanned each hybridized array using an Affymetrix 428 Array Scanner (MWG Biotech, High Point, NC, USA) to produce a digitized image of ‘red’ and ‘green’ fluorescence intensity data for each spot (*n* = 19 200) on each array (total of eight). We captured and exported red–green data from TIFF images using the image analysis software ScanAlyse (v2.5; Michael Eisen, Stanford University). We used these data to assess general hybridization quality from standard diagnostic plots applicable to two-colour arrays (Yang & Paquet, 2005).

Data normalization included two preprocessing steps. First, we corrected against nonspecific (background) hybridization in each channel using the ‘adaptive’ method of Smyth (2005). Second, we added to the background-corrected intensities a positive constant (= 50) to dampen spurious variation in log-ratios, particularly at low intensity spots. We accounted for further intensity dependent biases as well as spatial biases in hybridization signal by fitting loess (locally weighted) regressions (cf Cleveland & Devlin, 1988) through *M* vs. *A* plots for each print-tip group (*n* = 48) on each array (where *A* = log intensity = log_2_√WT•AN), and used as normalized *M*-values the residuals from these regressions. Further, we scale normalized between arrays so that each array had the same average intensity (Smyth, 2005).

We estimated the fold-change in expression and its standard error for each gene by fitting a linear regression to the normalized expression data using least squares (Smyth, 2004; Yang & Speed, 2003). The linear model incorporates the dye-swap design as a covariate, and uses information from duplicated spots, following the estimation of a common gene-wise expression value (Smyth *et al*., 2005). As recommended (Smyth, 2004), we applied a Bayesian smoothing procedure to ‘shrink’ the estimated standard errors, and from the ratio of *M*-values to their standard errors calculated a moderated *t*-statistic for each gene (Smyth, 2004). We identified significant genes by their associated *P*-value, following an adjustment for multiple comparisons which strongly controls for false discovery rate (*q*-values; Benjamini & Hochberg, 1995).

All preprocessing, normalization, and fold change calculations were performed using the software limma (v2.4.7 Smyth, 2005) available through the Bioconductor project (Gentleman *et al*., 2004).

### Bioinformatic characterization of candidate genes

Following identification of differentially expressed genes from the arrays, we used the Blast family of search functions (http://www.ncbi.nlm.nih.gov) to detect homology between probe cDNAs of interest and the honey bee genome (versions 2.0–4.0; http://www.ncbi.nlm.nih.gov/genome/guide/bee), as well as to detect homology between honey bee gene sequence and that of other organisms. Probe sequence was mapped to its genomic locus, where known, using NCBI's Honey Bee Map Viewer. We also queried the molecular function of candidate genes with reference to published information on these genes or their homologues.

## Supplementary material

Table S1 Summary plots of normalized expression data for a) brain and b) abdomen experiments. Comparative boxplots of *M*-values by array and experiment, and *MA*-plots of combined data, where *M* = log2 ratio and *A* = log2 intensity. Comparative boxplots show that the spread of *M*-values are roughly similar between arrays, and composite *MA*-plots are roughly symmetric about *M = 0*.

This material is available as part of the online article from http://www.blackwell-synergy.com
