# Supplementary figures and images for: Towards a molecular definition of worker sterility: differential gene expression and reproductive plasticity in honey bees
Source: Insect Mol Biol. 2006 Oct 1;15(5):637–44. doi: 10.1111/j.1365-2583.2006.00678.x (PMC1847478; doi:10.1111/j.1365-2583.2006.00678.x)

## Slide 1
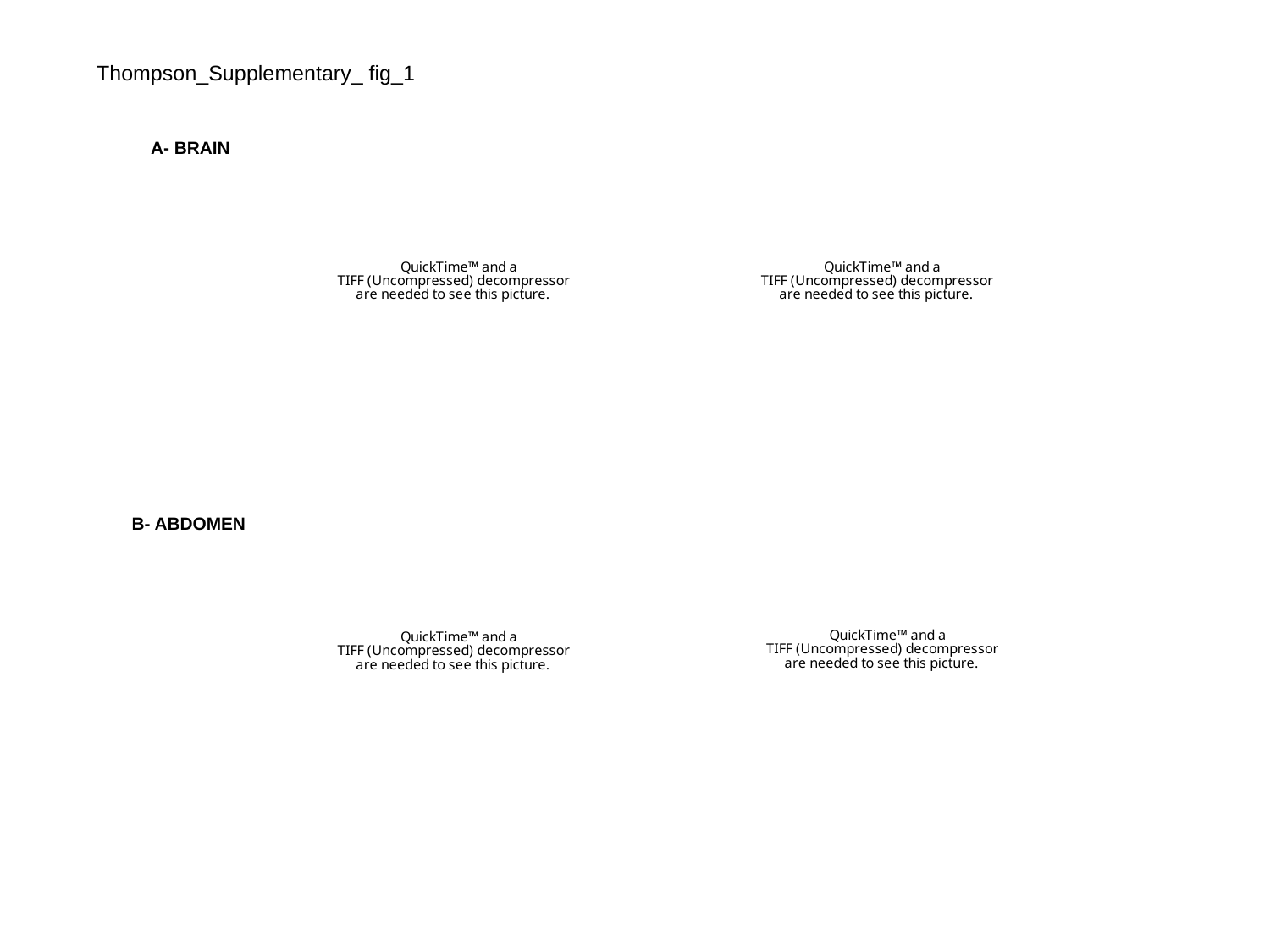

Thompson_Supplementary_ fig_1
A- BRAIN
B- ABDOMEN

Supplement: Table S1 — Summary plots of normalized expression data for a) brain and b) abdomen experiments. Comparative boxplots of M-values by array and experiment, and MA-plots of combined data, where M = log2 ratio and A = log2 intensity. Comparative boxplots show that the spread of M-values are roughly similar between arrays, and composite MA-plots are roughly symmetric about M = 0. [file imb0015-0637-ts1.ppt]
